# A Novel Case of Penile Gangrene in a Patient Treated with Ibrutinib for Chronic Lymphocytic Leukemia

**DOI:** 10.1155/2016/6980198

**Published:** 2016-11-23

**Authors:** William Paul Skelton IV, Neeka N. Akhavan, Zachary A. Taylor, Thu-Cuc Nguyen, Hassan Hassan, Tabitha N. Townsend, Prajwol Pathak, Gaurav Trikha, Nam H. Dang, Long H. Dang, Azka Ali

**Affiliations:** ^1^Department of Internal Medicine, University of Florida, Gainesville, FL, USA; ^2^Department of Internal Medicine, University of Central Florida, Orlando, FL, USA; ^3^Cancer Specialists of North Florida, Saint Augustine, FL, USA; ^4^Division of Hematology and Oncology, Department of Internal Medicine, University of Florida, Gainesville, FL, USA

## Abstract

*Introduction*. Ibrutinib is commonly used for the treatment of patients with CLL in either first-line or relapsed/refractory settings.* Case Presentation*. We present the case of a 74-year-old Caucasian man with CLL who presented with penile gangrene upon initiation of ibrutinib treatment. Our case is the first showing the complication of penile gangrene associated with ibrutinib use. The gangrene was self-limited upon discontinuing ibrutinib.* Conclusion*. Our finding describes a very rare yet important adverse event associated with ibrutinib use.

## 1. Introduction

Chronic lymphocytic leukemia (CLL) is characterized by monoclonal proliferation of CD5 positive B cells, and this phenotype also has coexpression of CD19, CD5, and CD23 markers [1, 2]. The most common deletion in CLL, which is associated with 50% of genotypes, involves chromosome 13q14, and this occurs at the stem cell level [3, 4].

Initiation of CLL treatment depends on the stage of disease. Asymptomatic, early stage patients should be observed without therapy unless there is evidence of disease progression [5]. Treatment with chlorambucil or chlorambucil plus prednisone did not increase survival in early stage CLL, and use of single-agent chlorambucil has also been shown to be effective to reduce toxicity and cost [6, 7]. Combination therapies such as fludarabine, cyclophosphamide, and rituximab regimen (FCR) improved progression-free survival and overall survival in physically fit, treatment naïve patients [8]. Other therapeutic first-line or relapse options include bendamustine, alemtuzumab, ofatumumab, or high dose corticosteroids [9]. More recently, targeted therapies against several tyrosine kinase inhibitors involved in the B cell signaling pathway in CLL cells have been studied and approved for treatment of CLL.

Ibrutinib is a Bruton's tyrosine kinase (BTK) inhibitor that effectively stops downstream survival pathways including ERK1/2, PI3 K, and NK-kB and induces B cell apoptosis [10]. The RESONATE-2 trial compared ibrutinib versus chlorambucil in treatment of naïve patients, and ibrutinib had improved outcomes in progression-free survival, overall survival, and response rate, as well as improvement in hematologic variables [11].

Ibrutinib has been associated with a higher frequency of remissions in relapsed or refractory CLL in a phase 1b-2 multicenter study by Byrd et al. [12]. Major side effects reported in the study were grades 1-2 and mainly included diarrhea (40%), upper respiratory infection (28%), fatigue (24%), cough (26%), arthralgia (23%), and rash (23%). A majority of adverse events resolved without interrupting therapy. Adverse reactions of grade 3 or above occurred early in the course of therapy and included pneumonia (12%) and dehydration (5%) [12].

A summary of reported adverse events from FDA-approved targeted therapies showed that frequency of all grades rash associated with ibrutinib was 16–28%, but the frequency of adverse event of grade 3 or above rash was 0% [13]. There are no known reported cases of skin necrosis or necrotic lesions of the skin associated with ibrutinib. Here, we present the case of penile gangrene associated with ibrutinib use encountered at the University of Florida Health Hospital.

## 2. Case Presentation

**Figure 1:**
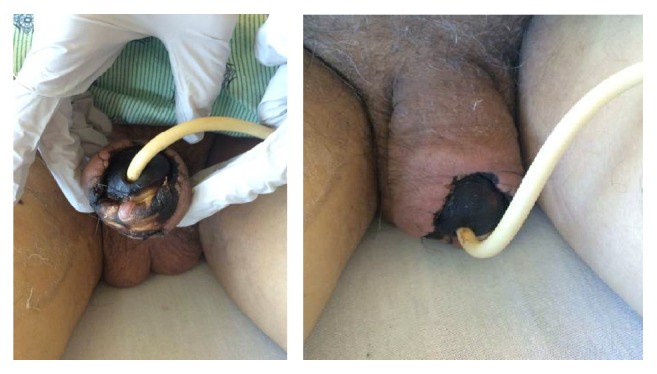
Gangrene of glans penis.

A 74-year-old Caucasian man with history of CLL (del 17p, del 13q) was initiated on ibrutinib therapy after experiencing an adverse reaction to the first cycle of R-CHOP. One month later, he presented to his primary care clinic with the complaint of a tender, discolored lesion of the glans penis. He was initially treated with acyclovir due to concern for Herpes Simplex Virus; however the lesion continued to worsen, which led to difficulty with urination. He was admitted to the hospital for further evaluation and management. The patient denied any history of sexually transmitted infection, condom catheter use, trauma to the penis, or unretractable foreskin. Past medical history was significant for type II diabetes mellitus and hypertension, but negative for known atherosclerotic disease. On examination, he was found to have an uncircumcised penis with a necrotic lesion of the glans penis with minimal sensation (Figure 1). There was no evidence of phimosis or paraphimosis.

**Figure 2:**
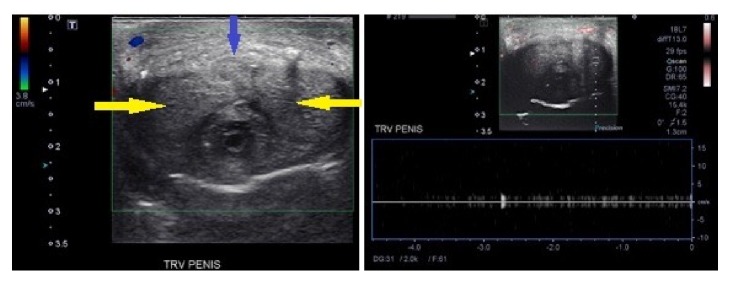
Penile ultrasound Doppler showing no demonstrable arterial flow within the heavily calcified penile arteries (yellow: cavernosal; blue: dorsal).

**Figure 3:**
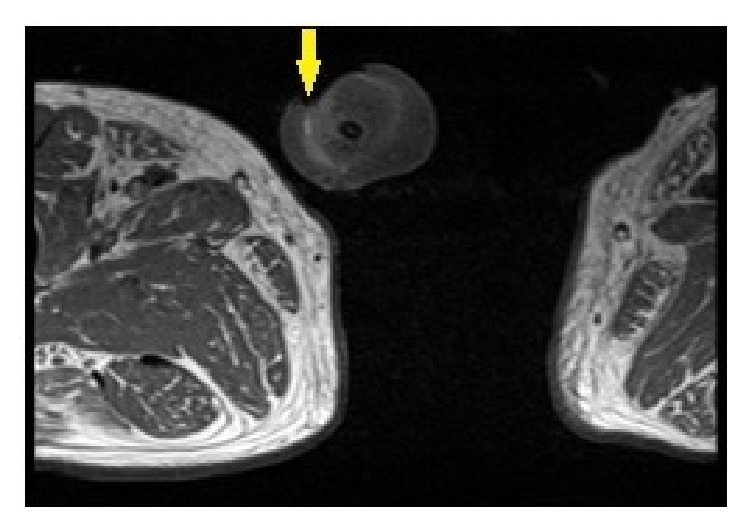
MRI pelvis showing T1 hyperintensity surrounding the glans penis, consistent with necrosis of the tip of the glans penis.

**Figure 4:**
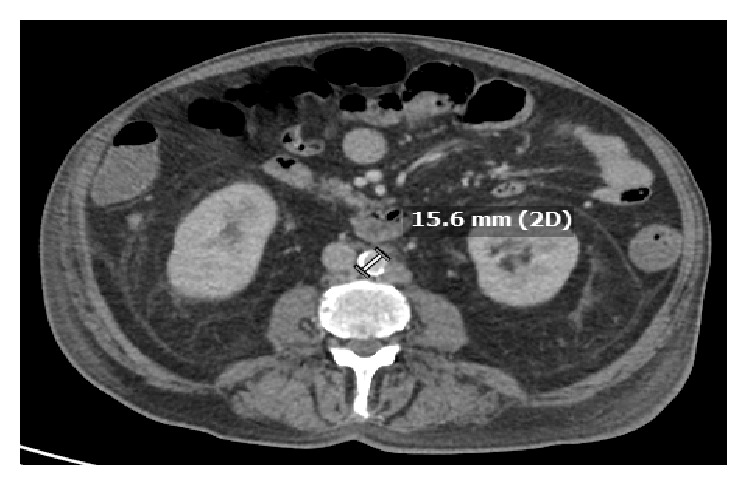
CT abdomen/pelvis with IV contrast showing the abdominal aorta measuring 15.6 mm at narrowest point.

At the time of presentation, white blood cell count was found to be 9,300/microliter. Penile ultrasound revealed no appreciable arterial flow in the penile arteries (Figure 2). Pelvic MRI showed minimal enhancement of the skin overlying the glans penis consistent with necrosis; there was no evidence of mass lesions of the penis (Figure 3). HSV serologies were negative. A workup for vasculitis was initiated; however ANCA, rheumatoid factor, cryoglobulins, and anticardiolipins were all negative. Workup for atherosclerotic disease was carried out with arterial Doppler of bilateral lower extremities which did not show evidence of arterial insufficiency. A CT scan of the abdomen/pelvis was negative for narrowing of the abdominal aorta (which could predispose to penile necrosis), as its narrowest point was 15.6 mm (Figure 4).

In this case, onset of symptoms after the initiation of ibrutinib supported a potential association. Moreover, he did not have any underlying risk factors for penile infection, trauma, or a history of phimosis or paraphimosis. Ibrutinib was discontinued as an alternative etiology was not discovered. Despite a comprehensive workup, no alternate etiology was found to explain penile necrosis, suggesting likely ibrutinib related adverse effect. Our urology colleagues were involved in the patient care and recommended allowing for autoamputation of the glans penis. A suprapubic catheter was placed to allow for bladder drainage during this process. After stabilization, the patient was discharged home. He did not experience any bleeding episodes on ibrutinib. Upon follow-up in the clinic, the gangrene had not progressed further.

## 3. Discussion

The association between ibrutinib use and penile gangrene has never been reported in the literature. Regarding the patient's comorbidities of hypertension and type II diabetes mellitus, they were well-controlled, as his HgbA1c was 5.6% and his systolic blood pressures had been well-controlled in the 120 s–130 s throughout his hospital course. Arteriosclerosis was also likely not an etiology of the penile gangrene because of his unremarkable lower extremity Doppler ultrasound, normal lactate, and having no personal history of cardiac events or coronary artery disease. There was also no evidence of infiltration of extramedullary CLL into the penis from the pelvis MRI (Figure 3). Further, the patient initially responded very well to ibrutinib, with his cervical adenopathy resolving after initiation of treatment.

Therefore, the most likely differential diagnosis in this case to explain the penile gangrene is a potential adverse event from ibrutinib use, with the onset of the necrosis coinciding with the initiation of treatment. Upon discontinuation of ibrutinib, the gangrene was self-limited.

Penile gangrene may be dry, infective, or vascular, and distinction is essential as management is different for each case. In setting of infection, an infection can be identified in over 90% of the cases. Dry gangrene, as in this patient, is classically vascular in origin; see common causes of dry (noninfectious) penile gangrene [14] as follows:

Diabetes mellitus
End stage renal disease
Tourniquet syndrome
Priapism
Venous thromboembolism
Anticoagulant therapy
Heroin injection into the femoral vessels

The challenging part of this case is that the definitive way to diagnose ibrutinib as the underlying cause was a biopsy of the glans penis. However, given that the patient had no demonstrable arterial flow within his heavily calcified cavernosal and dorsal penile arteries, there was concern that he would have significant troubles with wound healing after penile biopsy. After consultation with our urology colleagues, it was determined that we would not proceed with penile biopsy (as this would potentially cause increased harm without necessarily changing management). Thus, no surgical intervention was recommended, as the glans was allowed to autoamputate over time.

As noninfectious gangrene is precipitated by an ischemic event, it is unclear through which mechanism ibrutinib could have precipitated this event. We postulate that the etiology in this case is potentially multifactorial and ibrutinib's ability to induce B cell apoptosis may have contributed to this event as the timeline of symptoms supports this association.

## 4. Conclusion

To our knowledge, this is the first case linking ibrutinib to a complication of penile gangrene. Prior to initiation of ibrutinib in patients with CLL, a thorough urogenital history should be obtained prior to initiation of ibrutinib. We recommend if this side effect is encountered to discontinue therapy, to rule out other causes of noninfectious gangrene, and to consider switching to an alternative regimen. Reporting rare adverse drug events is important to increase our knowledge, especially in the field of oncology where many novel therapies are currently being developed, and to that end, further investigation and monitoring of side effects are warranted.
